# Hepatitis B in the Greater San Francisco Bay Area: an integrated programme to respond to a diverse local epidemic

**DOI:** 10.1111/j.1365-2893.2010.01382.x

**Published:** 2011-04

**Authors:** RG Gish, SL Cooper

**Affiliations:** 1 Division of GI Hepatology, University of CaliforniaSan Diego, CA, USA; 2 Division of Hepatology, San Francisco Center for Liver DiseaseSan Francisco, CA, USA; 3 Department of Medicine, California Pacific Medical Center and Research Institute, and Sutter Pacific Medical FoundationSan Francisco, CA, USA; 4 Department of Transplantation, California Pacific Medical Center and Research Institute, and Sutter Pacific Medical FoundationSan Francisco, CA, USA; 5 Liver Immunology Laboratory, California Pacific Medical Center and Research Institute and Sutter Pacific Medical FoundationSan Francisco, CA, USA

**Keywords:** Asians and Pacific Islanders, at-risk populations, cancer surveillance, chronic hepatitis B, culturally targeted screening, disease surveillance, hepatitis B virus, hepatocellular carcinoma, high-risk populations, screening, vaccination

## Abstract

Although chronic hepatitis B (CHB) affects approximately 2 million United States residents, there is no systematic screening of at-risk individuals, and most remain unaware of their hepatitis B virus (HBV) infection. Unmonitored and untreated, CHB results in a 25–30% risk of death from liver cancer and/or cirrhosis, inflicting an increasing healthcare burden in high-prevalence regions. Despite high prevalence in immigrant Asians and Pacific Islanders, among whom CHB is a leading cause of death, community and healthcare provider awareness remains low. Because safe and effective vaccines and effective antiviral treatments exist, there is an urgent need for integrated programmes that identify, follow and treat people with existing CHB, while vaccinating the susceptible. We describe an extant San Francisco programme that integrates culturally targeted, population-based, HBV screening, vaccination or reassurance, management and research. After screening over 3000 at-risk individuals, we here review our operational and practical experience and describe a simple, rationally designed model that could be successfully used to greatly improve the current approach to hepatitis B while ultimately reducing the related healthcare costs, especially in the high-risk populations, which are currently underserved.

## Introduction

**Fig 1:**
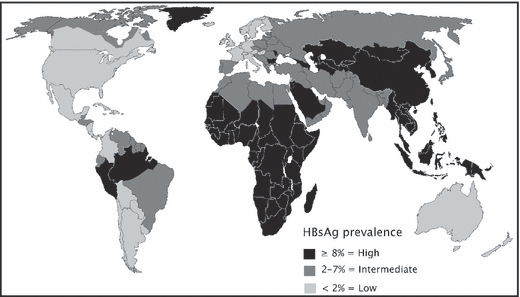
Geographical distribution of hepatitis B virus (HBV) infection – worldwide, 2006. For multiple countries, estimates of prevalence of hepatitis B surface antigen (HBsAg), a marker of chronic HBV infection, are based on limited data and might not reflect current prevalence in countries that have implemented childhood hepatitis B vaccination. In addition, HBsAg prevalence might vary within countries by subpopulation and locality. Source: Centers for Disease Control and Prevention (CDC) Traveller's health; yellow book. Atlanta, GA: US Department of Health and Human Services, CDC; 2008.

Chronic hepatitis B (CHB) is a major public health problem, affecting an estimated 400 million people worldwide [1,2], and at least 1.5–2 million people in the United States (US) [3]. The Hepatitis B Foundation (HBF) calculates that the US CHB prevalence is substantially larger than the previously accepted estimate of 1.25 million, because the highest-risk populations are under-represented in surveillance studies that have generally not accounted for the large and ongoing influx of foreign-born individuals from countries with moderate and high hepatitis B virus (HBV) prevalence (Fig. 1), and because a large percentage of chronically infected individuals remain undiagnosed [4]. Using census and current HBV prevalence data, HBF estimates the current burden of CHB in the US to be approximately 2 million people.

Despite these high numbers, even in the US there is no systematic nationwide screening of at-risk individuals, and most people with CHB remain symptom-free and unaware that they are infected until a late disease stage when they develop clinically apparent hepatocellular carcinoma (HCC) and/or cirrhosis. Without medical monitoring and treatment, the risk of HCC and cirrhosis, with sequelae of liver transplantation or death, is 25–30% [5–9]. Modelling the burden of CHB worldwide, the Centers for Disease Control and Prevention (CDC) estimated that in the year 2000, 620 000 persons died from HBV-related causes, with 580 000 (94%) from CHB-related cirrhosis and HCC [10]. These estimates were almost certainly conservative.

The disease spectrum of CHB is highly variable. At one extreme, some persons experience inactive infection (no hepatitis) throughout a lifetime, while at the other some develop cirrhosis and HCC during childhood. Between these extremes are many intermediates where there are irregular cycles of disease activation (‘flares’ in hepatic enzymes with liver inflammation, injury and fibrosis) and dampening. In developed countries, CHB can be effectively treated with antiviral drugs when individuals have access to informed healthcare providers and adequate healthcare coverage.

The acquisition of HBV can largely be prevented. Vertical transmission from mother to child can be arrested in up to 95% of cases by prompt neonatal vaccination and infusion of hepatitis B immune globulin (HBIG) [11]. Universal neonatal HBV vaccination, introduced in the US in 1991, has resulted in immunity in a high proportion of young children [12]. Horizontal transmission can also be prevented by vaccination, which is highly effective [13] and unequivocally safe, although the use of vaccination by at-risk adults remains disappointing.

Because safe and effective vaccines and therapies exist, there is an obvious humanitarian need to identify, follow and, when necessary, treat people with existing CHB, while also vaccinating their contacts to prevent transmission. For efficient case identification, there is an imperative to systematize this task by moving towards regional and national programmes that deploy targeted screening, with no socioeconomic repercussions for people who test positive.

Hepatitis B virus has particularly high prevalence in Asians and Pacific Islanders (API), most of whom become chronically infected at birth or in childhood [14]. It is estimated that API constitute more than half of the global reservoir [10]. In recent years, screening programmes in New York City, Philadelphia, Atlanta, Chicago and California have reported 10–15% prevalence of CHB among immigrant API [15–19]. In regions with high API population density, the management of patients with cirrhosis and HCC will produce an increasing healthcare burden without prompt, effective intervention. However, there is still no nationally coordinated programme that systematically addresses the identification and requirements of Americans living with CHB, especially in high-prevalence populations. Unfortunately, the National Institutes of Health Consensus Development Conference on the management of HBV in October 2008 devoted little attention to screening, liver cancer surveillance, access to healthcare services, or cultural and other barriers inhibiting awareness, testing, and accrual of knowledge about the necessity for monitoring and how to obtain well-informed medical management.

In early 2010, the Institute of Medicine (IOM) released an in-depth report entitled *Hepatitis and Liver Cancer: A National Strategy for Prevention and Control of Hepatitis B and C* [20] which noted the urgent need to address these diseases and discussed three major factors currently impeding efforts to prevent and control them:

- Lack of knowledge and awareness about these diseases among healthcare and social-service providers.
- Lack of knowledge and awareness about these diseases among high-risk populations, as well as the general public and policy-makers.
- Insufficient knowledge of the extent and seriousness of this problem resulting in inadequate public resources being devoted to prevention, control and surveillance programmes.

The IOM report gave detailed recommendations for improving surveillance, increasing knowledge and awareness among both healthcare practitioners and the public, improving immunization programmes and expanding viral hepatitis services. The report detailed major gaps in these areas with at-risk populations and foreign-born residents. Noting that nearly half of the US foreign-born population comes from HBV-endemic countries, a strong recommendation was made for culturally appropriate screening programmes and related services for this population.

Efforts to address all of these concerns have so far consisted mainly of relatively small local or regional US programmes targeting high-risk populations. A recent nationwide survey identified only a possible 55 community-based screening and vaccination programmes in the US, with the majority found in California (23 programmes) and the mid-Atlantic region (14 programmes) and the rest scattered through the Midwest (6 programmes), New England (3 programmes), Hawaii (2 programmes), Texas (2 programmes), Colorado (1 programme), Washington (1 programme) and Louisiana (1 programme) [21]. Two programmes did screening in multiple states. Although the lack of response from 20 programmes indicates that they may no longer be operational, the researchers confirmed that 32 of these programmes were currently screening or had screened within the last year. In addition to screening and vaccination, 74% of the programmes provided HBV education and 71% treatment referrals. Only 29% directly provided HBV treatment.

Two previously established programmes can serve as examples of projects that have been effective in providing HBV education and screening. The nonprofit Asian Liver Center (ALC) at Stanford University's Jade Ribbon Campaign (JRC) was established in 2001 to raise awareness and provide information about HBV and liver cancer to both the API community and health professionals. The JRC incorporated access to vaccination but not to treatment. In 2005, the Asian American Hepatitis B Programme (AAHBP), a collaboration of community groups and academic and community health centres, was launched to provide hepatitis B screening, vaccination and treatment to the API population in New York City [18]. AAHBP also provides educational programmes to increase awareness of HBV infection among API communities.

In November 2006, San Francisco Resolution 650-06, proposed by current California State Assemblywoman Fiona Ma, established the goal of universal HBV screening and vaccination among the approximately 240 000 API and other high-risk residents of San Francisco. The San Francisco Department of Public Health (SFDPH) and the AsianWeek Foundation (AWF) coordinated this *SF Hep B Free* program with major citywide healthcare providers and community organizations. A steering group comprised of SFDPH, AWF, and the ALC guided the establishment of a working group to develop the programme which included our non-profit San Francisco institutions, the California Pacific Medical Center (CPMC) and its independent affiliate, the Sutter Pacific Medical Foundation (SPMF). CPMC/SPMF (CPMC) was asked to play a major role on the basis of established expertise, infrastructure and history of integration into the multiethnic San Francisco communities. Major efforts are now underway by public health departments, the American Liver Foundation, the CDC, the American Association for the Study of Liver Disease, the HBF, and other parties to tackle the US HBV burden. The CPMC experience shows both the highlights and pitfalls of an integrated approach for detecting, preventing and managing HBV infection in a US region of high endemicity. The information may help refine plans for developing effective regional HBV healthcare strategies.

## Overall goals of a chronic hepatitis B integrated programme

An effective, integrated response to hepatitis B in San Francisco, the Greater Bay Area and northern California requires increased awareness by healthcare professionals, policy-makers and the general public about the importance of targeted screening and testing for infection. Educational programmes thus need to be directed towards all these groups, with a particular need for HBV-specific medical education directed to healthcare providers, as emphasized by recent surveys showing that 30–55% would order the wrong test to screen for HBV infection [22]. The goals of our citywide and regional programme are to:

- Raise awareness among healthcare providers regarding those populations (such as API) that are at high risk for HBV infection and the importance of proactive testing.
- Increase awareness and knowledge among the general public and healthcare professionals of the prevalence, natural history and effective management of CHB.
- Highlight the need for an integrated approach to management once HBV infection has been identified, including:
  - Liver cancer surveillance;
  - Monitoring for disease activation;
  - Treatment of patients who fit current evidence-based criteria.
- Raise awareness in the community that HBV infection is preventable through vaccination and, when active, is treatable with safe, effective antiviral agents.
- Emphasize the need for API to become aware of screening, vaccination, disease surveillance and treatment programmes and of how to gain access to them.
- Identify and deconstruct barriers to screening.
- Increase vaccination rates among those who are susceptible.
- Correct common misperceptions about HBV infection, vaccination and treatment.
- Outline specific areas where additional research is needed to guide best practices and how this coordinated programme can add to those research efforts.

## Specific objectives of screening programme

A strategically designed screening programme can substantially lessen the impact of CHB, especially in a high-risk immigrant population such as that which exists in the Greater San Francisco area. CPMC's adult HBV screening programme focuses on the four interwoven areas of education, diagnosis, prevention and treatment with the following key objectives:

In at-risk persons, to:

- Identify susceptible individuals and implement vaccination;
- Identify and inform individuals with immunity;
- Identify and manage infected individuals.

To conduct surveillance for:

- Disease activation in individuals whose disease has been quiescent;
- Primary liver cancer (HCC).

To initiate treatment in appropriately identified individuals and survey for:

- Treatment success;
- Treatment failure because of either viral resistance or lack of compliance.

## Chronic hepatitis B in San Francisco, the Bay Area and northern California

### Epidemiology and context of chronic hepatitis B in the San Francisco Bay Area

Chronic hepatitis B is particularly prevalent in San Francisco, the Bay Area and northern California and is likely to remain so because of continuing high immigration from areas of high HBV endemicity, particularly Asia and the Pacific Islands (Fig. 1). API comprise approximately 32% of the population of San Francisco [23], with an estimated 25% (approximately 60 000 people) born in Asia or the Pacific Islands. According to a report from the SFDPH and CDC, 84% of individuals with confirmed CHB reported in San Francisco in 2006 were API, 80% of whom were born outside the United States [24].

The clinical consequences are clearly apparent. In California as a whole, HCC is one of the five most common cancers in Cambodian, Chinese, Filipino, Hawaiian, Korean, Laotian, Pacific Islander and Vietnamese men [25]. Commensurately, in the Greater Bay area, HBV-related HCC is a leading cause of death among API, ranking as the second leading cause of cancer death among Chinese and Vietnamese men, the third among all API men and Vietnamese women, and the fourth among Korean men [26]. The range probably reflects the differential prevalence of CHB in different API subgroups. CHB and liver cancer rates are, in fact, among the greatest health disparities between API and Caucasian Americans, with the incidence of HCC in API men more than three times higher than in Caucasian men [26]. Despite such alarming statistics, because of low awareness of risk and paucity of symptoms many individuals do not know that they are infected. The consequences include increased risk of transmission, as well as failure to seek monitoring and treatment, leading to substantially increased risk of disease progression.

Access to healthcare among the API populations of California may also be limited. For instance, the town of Merced, located in the agricultural San Joaquin Valley, is home to more than 6000 Hmong people [27], a group ancestrally from southern China [28]. Because of a language barrier, Hmong individuals experience difficulty obtaining healthcare and understanding interactions with healthcare professionals during screening and physical examinations. Studies have shown that, in general, Asians who lack fluency in English are less likely to receive adequate preventive healthcare [29].

Coinfection with HBV and human immunodeficiency virus (HIV) and/or hepatitis C virus (HCV) among API Americans is less common than HBV monoinfection but in the Bay Area, HIV and HCV are also highly prevalent. Coinfected individuals are likely to have higher serum HBV DNA levels and experience faster progression to cirrhosis and liver cancer [30]. Accurate rates of coinfection with HBV are unclear at this time and warrant investigation. In the CPMC programme, when a person with HBV is identified, a medical professional will ask screening questions to identify those at risk for HIV and HCV, and subsequent testing will be ordered, if indicated and feasible, depending upon healthcare coverage. It is important to be aware of fears about the possible consequences of testing positive in a private insurance, managed healthcare system, and for members of the healthcare team to discuss these issues with patients.

## Target populations for hepatitis B screening

Recommendations for HBV screening are based on the population groups at risk. In addition to API Americans, other high-risk groups living in San Francisco and the Bay Area who should and are being screened for HBV infection include the following [31]:

- Family members of individuals who have HBV infection.
- Individuals born in other areas with intermediate to high prevalence for HBV infection.
- Pregnant women (currently mandated in California).
- Women with a history of many sexual partners.
- Individuals with history of sexual contact with HBV-infected persons.
- Individuals who inject drugs.
- Correctional facility inmates.
- Men who have sex with men.
- HIV-infected and/or other immune-compromised individuals.
- Individuals with chronic liver disease.
- Haemodialysis patients.

## Public health response to hepatitis B

San Francisco, the Greater Bay Area and similar regions require a comprehensive plan for detecting and managing local HBV infection which implicitly includes provision of access to healthcare. The task of developing a unified programme is hampered by a series of factors, including social and cultural diversity between and within the high-risk populations. A central element of our local public health plan is a coordinated screening programme directed towards individuals at greatest risk of HBV infection and transmission. Those found neither infected nor immune should undergo vaccination, which we are encouraging and incentivizing in our CPMC programme by providing free service and vaccine. In addition, feasible models for the appropriate management of individuals who are already infected need to be more effectively developed and implemented.

A public health response to hepatitis B in San Francisco, the Greater Bay Area and northern California should include the following components:

- Educational programmes that inform high-risk individuals about screening, vaccination and treatment options;
- Implementation of culturally sensitive HBV screening and vaccination programmes in API and other ethnic communities that would identify individuals with CHB, help them obtain appropriate management including cancer surveillance and treatment, and explain the indications, benefits, risks and side effects associated with the various medications [16];
- Use of electronic registries by local health departments and healthcare systems to provide services to individuals with CHB, including ability to put HBV-infected individuals in contact with appropriate prevention and care services.

## The CPMC screening programme: infrastructure, logistics and results to date

### Personnel

As part of the *SF Hep B Free* initiative, CPMC has committed to screen at least 10 000 local API at no cost to the subject. Practical implementation of the CPMC HBV screening programme has principally been orchestrated by a coordinator who is the only salaried member of our programme. Multilingual volunteers including nurses, SPMF research laboratory scientists, SPMF Liver Center clinical administrative personnel, a programme consultant and supervised phlebotomy students were deployed to staff each screening site. Certain key personnel, including the programme coordinator, were present for each screening event. A hepatologist was also present at most events. Full details on screening site personnel and their duties are available in the supplementary materials and on our website at: http://www.cpmc.org/advanced/liver/physicians/default.html.

### Funding

Funding for the CPMC HBV screening programme was provided by unrestricted charitable grants from CPMC Foundation and from Gilead Sciences. Screening tests for hepatitis B surface antigen (HBsAg) and anti-HBs were performed by Quest Diagnostics (Madison, New Jersey) under special contract. Because of cost, we omitted testing for the hepatitis B core antibody.

### Creating awareness and providing education in our multilingual community

Developing a cost-effective HBV awareness and education programme for API and their healthcare providers has been challenging, especially within our budget. The objective of encouraging testing with appropriate follow-up for API requiring vaccination, monitoring or management remains an evolving work in progress. CPMC, in concert with the *SF Hep B Free* programme, has coordinated a series of public notices and education efforts. These efforts have included banners on buses and bus-stop structures, billboards, information published in English and Asian language newspapers, radio broadcasts and television exposure. In the aggregate, these have promoted community awareness of the overall programme, while we focused awareness on specific issues via media events, news articles and press releases.

Some of the initial efforts were not cost effective. For example, early in the CPMC programme the authors recorded public service announcements (PSAs) broadcast on local radio stations. The PSAs delivered an educational message and an appeal to get tested (http://www.cpmc.org/advanced/liver/physicians/default.html). However, even with negotiated pricing, a 1 minute radio spot, broadcast daily for 1 week, cost $10 000 USD, prohibiting this as a regular medium. Finding local media willing to broadcast such PSAs at little or no cost would be important for programmes wishing to use this approach to raising awareness.

Promotional posters have been developed specifically by our team and deployed in and around the four main CPMC campuses in San Francisco, supplemented by campus-wide e-mailed newsletters. The content delivered awareness and encouraged testing of at-risk individuals and their family members. Particularly effective has been the combination of education and screening events that we have conducted at higher educational establishments and at venues of natural congregation within the API community, including community centres and clinics, churches and restaurants. More recently, we extended the same strategy to workplaces employing significant numbers of API. Such events have resulted in approximately 50–100% of attendees being tested. API street fairs and festivals achieve lower proportional testing rates but high aggregated numbers, and permit economically efficient awareness and education opportunities, including within families. Other ongoing strategies include high school-based HBV education seminars with competitions and prizes for students designing the most innovative and communicative posters.

Cultural alignment of all components of the programme has been complex. In an effort to tackle this at a basic level we generated printed materials reflecting the major local Asian languages and recruited voluntary programme members who are multilingual. On the *SF Hep B Free* website (http://www.sfhepbfree.org), there are currently links to very brief videos that explain the importance of screening, vaccination and treatment in English (http://www.youtube.com/watch?v=3k-O3DE2HfI&feature=related), Mandarin (http://www.youtube.com/watch?v=BKTdMiHvX_U) and Cantonese (http://www.youtube.com/watch?v=pBny8WvDkSA). To improve this aspect of our programme, we are considering creating additional video presentations in all of the common Bay area Asian languages which could be made freely available online. The presentations would provide a concise overview of the need for screening and the resources available for this at our locations and those of our city screening partners. The videos would conclude with a web address linking to more information in that particular language, a link that would take the viewers to the materials we have already produced. To tackle this more deeply would probably require more resources than are currently available. This is an area requiring more research.

More recently, we have been able to minimize media expenditure as a result of our CPMC programme being conducted in conjunction with the citywide *SF Hep B Free* initiative. The latter has gradually gained momentum and is now providing most of the advertising. The programme has deployed culturally attuned newspaper advertisements, posters, and placards on public transport. In addition, there is now a contracted marketing organization that helps position news and press releases that are linguistically and culturally focused and put forth in a timely fashion before each screening event.

The authors and their specialist colleagues also conduct CME events for API healthcare providers within CPMC and throughout the city, although such events typically attract only low numbers of key individuals. With other physician members of the medical protocol group of the *SF Hep B Free* programme, the authors helped develop a simple flow chart aimed at promoting appropriate HBV testing by community API healthcare providers and facilitating test interpretation and subsequent management decision-making. The flow chart is available at: http://www.sfhepbfree.org/files/education_clinicians/Flowchart_Diagnostic_revised2009.pdf. A proposal by us for the California State Medical Board to mandate that physicians should successfully complete a basic hepatitis B medical education course as a prerequisite for license renewal (akin to an earlier pain management course) failed to gain traction.

### Screening results to date

In the first 24 months, 3120 people were screened at forty sites encompassing eight different types of venue. These included higher educational establishments with large numbers of Asian students (*n* = 1412 people screened at this type of site), Asian street festivals and fairs (*n* = 922), Asian restaurants (*n* = 324), churches (*n* = 35), a community centre (*n* = 41), the CPMC hospital campuses (*n* = 377) and the CPMC liver clinics (*n* = 9). The majority of screening events were held during daytime hours.

Of the 3120 people screened, 2477 (79%) were identified as API. We identified 6% HBV prevalence among 1798 foreign-born (first generation) individuals, 2% prevalence among 365 second-generation API and 0% among third-generation API, although only 37 of the latter were screened. The proportion of already immune individuals was ∼54%, ∼58% and ∼43% in each generation, respectively. Test results automatically deploy into a secure and confidential database purchased from HKS Medical Information Systems (Omaha, Nebraska, USA) and dedicated solely to the HBV screening programme. At some screening events in API communities, fewer than 50% of attendees came forward to be tested, suggesting a significant possibility of sampling error in our dataset.

Screened individuals have been informed of their test results, given a simple explanation of the meaning of these results and advised regarding a course of action using letters mailed to their stated places of residence. Depending on the language information provided on their intake form, we have sent letters in English, Chinese, Vietnamese or Korean, where appropriate; samples of these letters are available both in the supplementary materials and at our website: http://www.cpmc.org/advanced/liver/physicians/default.html. In addition, telephone calls to every subject who tested HBsAg-positive have been conducted by the programme coordinator, the consultant and one of the authors (SC), supplementing the standard letter directed to the patient. Telephone follow-up has augmented successful contact and response, particularly among foreign-born individuals who failed to receive, open or understand the mailed letter. In some cases, we have been unable to confirm that positively screened subjects have been made aware of their infection status because of inability to contact them either directly or indirectly through an intermediary such as a primary care provider. To date, we have made personal contact with 73 of 116 (63%) people who tested HBsAg-positive, and we are continuing to attempt to follow up with the remaining 43 people.

Thus, to the greatest extent possible within this framework, those already immune to HBV have been informed of their protected status, and those susceptible to infection have been offered free vaccination at dedicated clinics. The outcome of our free vaccination programme is summarized in Table 1. Only 325 (23%) of the 1410 susceptible API have returned for vaccination to date. Because vaccination costs were not a factor, these data emphasize the need for even more resources to be devoted to a more culturally aligned education and call-back system to enhance complete follow-up for a full cycle of HBV vaccination. To this point, 193 (59%) of 325 initial vaccinees have completed the three-shot vaccination series. In 2010, we attempted to more deeply incentivize vaccination by charging an upfront fee of $60.00, which is fully refundable for individuals who complete the vaccination series.

**Table 1:** Free hepatitis B virus vaccination uptake by susceptible Asians and Pacific Islanders (January 2008 through December 2009)

Number screened	Number susceptible	Number of vaccinations		
		x1	x2	x3
3120	1410	325	254	193

### Management of HBV-infected API

The management of API found to be HBV infected (HBsAg+) has been greatly affected by the presence or absence of adequate healthcare coverage.

#### API with healthcare insurance

When healthcare coverage permits, persons newly identified with HBV have been offered follow-up consultation and laboratory testing, as well as HCC surveillance and antiviral treatment, when appropriate.

#### API without healthcare insurance

People who test positive for HBsAg but who do not have medical insurance coverage have posed an obvious problem in California. Referrals to a local county hospital have resulted in long delays (more than 1 year in some instances) before achieving access to specialist care, even for patients with active hepatitis B, exposing intrinsic deficiencies in California's private insurance, managed care-based system. Cultural attitudes and educational hurdles involving both patients and their primary healthcare providers amplify such healthcare deficiencies. In an effort to address this, all HBsAg+ uninsured patients have been offered free consultation with one of the authors (SC) at CPMC in which the implications and their options are discussed. They are also examined for the presence of clinically significant liver disease. As an initial option, they are offered applications for CPMC and SPMF Charity Care (two distinct programmes) and are assisted in completing the respective applications. The Charity Care programmes pay for a range of further investigations including liver tests and, when indicated, a sonogram. However, the virological tests that we consider the standard of care, particularly the HBV viral load, are assayed at external commercial laboratories, the costs of which are not covered by our charity care programmes. When the individual is able to pay out of pocket, quantitative HBV nucleic acid testing can be obtained at reduced cost, typically for about $120 per test.

Patients with elevated transaminases and/or viral loads are considered eligible for antiviral therapy and possibly for liver biopsy. For those requiring nucleos(t)ide analogue (NA) therapy who cannot afford to pay for the medications, applications have been made to pharmaceutical company patient assistance programmes which have been unanimously successful to date. No patients have yet been discovered with late-stage disease, including HCC, which would present an even greater challenge. A relatively new programme operated by the SFDPH called *Healthy San Francisco* (http://www.healthysanfrancisco.org/) provides an eventual avenue for care for the uninsured who reside in the city of San Francisco. It is available regardless of immigration status, employment status or preexisting medical conditions. All residents with an income at or below 500% of the Federal Poverty Level (in 2010, for one person $54 150 USD; for a family of four $110 250 USD) are eligible to enroll in the programme, and fees (both quarterly participant fees and point-of-service fees) are based on a sliding scale according to income. The programme is intended to make comprehensive healthcare services within the city of San Francisco accessible and affordable to all uninsured residents. In practice, some patients with active disease have found the programme difficult to navigate and their access to care has been stalled for long periods, indicating that refinements will be necessary. However, as our screening programme expands, *Healthy San Francisco* may become an increasingly honed and valuable resource for patients. In countries with national healthcare programmes, the rigours of obtaining hepatitis B management should prove less onerous for infected citizens.

The major benefits to a screened population are vaccination of ‘at-risk’ persons, the possibility of prompt evaluation and treatment of people with active hepatitis and/or incipient liver failure, and early identification and treatment of HCC. Currently, however, despite our programme, there is no system in place that provides timely universal care to all those who need it. In parallel with this screening programme, we are examining cost effectiveness and barriers to screening and healthcare access. The latter pose significant obstacles to programme success, not only for our programme but for all programmes with similar goals, particularly in the absence of a national healthcare programme.

Ultimately, the impact of implementing widespread screening strategies if a large number of patients do not have care or medication coverage is a fundamental question that extends beyond this manuscript and probes the core of the current healthcare debate in the United States. Is it ethical to screen for hepatitis B when a significant proportion of those affected do not have access to modern standards of care? We argue that hepatitis B satisfies criteria adopted by the World Health Organization for implementation of a screening programme [32]. We propose that identifying the extent of the hepatitis B problem and the associated clinical need may provide the most compelling case for healthcare provision and reform. In the interim, we are deploying the measures already discussed to provide care and to assist with medication coverage.

### Research

There are deficiencies in the evidence quantifying the true burden and natural history of hepatitis B in the US immigrant population. Using data from the screening programmes, a long-term follow-up programme carried out by CPMC in conjunction with city healthcare partners and SFDPH will begin tackling this. Adjunctively, molecular epidemiological studies will attempt to correlate the natural history of hepatitis B in this region of the US compared with the country of origin. These data will contribute substantially to the base of knowledge, particularly as there is a current dearth of information deriving from population studies in immigrants living in contemporary America.

## Possible challenges for similar programmes

We have identified certain obstacles that may present challenges in other areas seeking to establish similar screening programmes.

- First and foremost, our CPMC programme has had the considerable political and practical support and backing provided by the City of San Francisco's legislation, discussed earlier, that established the goal of implementing universal screening and vaccination for HBV infection among API and other high-risk residents. In addition, we were working as part of the SFDPH's *SF Hep B Free* programme. In other areas, particularly those where there is less awareness of the extent of HBV infection and the need for screening and treatment, such support may be lacking.
- As already mentioned, promotion of awareness about the need for screening and the existence of a screening programme can be costly. The CPMC programme had the advantage of the promotional efforts provided by the *SF Hep B Free* programme. In areas without similar initiatives, the full cost would be borne by the screening programme.
- In any given region, it will be necessary to identify the languages most commonly spoken so that materials in those languages can be obtained and made available. The CPMC programme will make materials it has developed freely available. However, in some areas there may be a need for materials in languages other than those commonly spoken in San Francisco, putting the burden on that community's programme of developing materials in additional languages.
- Our programme has relied heavily on volunteers, including a consultant, multilingual nurses, phlebotomy students and hepatologists willing to donate their time. Finding volunteers who have the necessary skills and are willing to donate their services repeatedly over time may be problematic.
- Our programme was only made possible through charitable grants that provided funding for a salaried programme coordinator and other expenses. Lacking a national health programme that would pay for screening services, the first step in other US communities may be to seek grants that will provide funding. In wealthy countries such as the US, we believe that reliance on grant funding for important healthcare initiatives is unnecessarily brittle.
- As discussed earlier, postscreening follow-up in line with modern standards of care, particularly for the uninsured, is vital. In regions lacking the type of charity care programmes in place at CPMC, or the type of citywide programme embodied in *Healthy San Francisco*, this may pose the largest obstacle.
- Last but not least, regions of relatively low HBV prevalence will require even more highly targeted programs of this type to maximize utility and cost effectiveness. Similar initiatives will be required. We anticipate, however, that such programs are justified and will remain cost effective owing to mitigating the substantial costs associated with managing the frequently serious complications in patients whose HBV has been unidentified.

## Priority action areas

The prevalence of HBV infection and related liver disease is increasing among API and other high-risk ethnicities in San Francisco, the Bay Area and similar US regions, primarily due to ongoing immigration from parts of the world where HBV is endemic. This creates the need for a coordinated plan of activities to reduce the impact on individuals already infected and to screen and identify individuals at increased risk of contracting HBV infection. Components of this coordinated plan would include prevention and education, screening and diagnosis, treatment and support, surveillance and research.

## Prevention and education

Hepatitis B virus transmission is preventable. The availability of hepatitis B vaccine led to an apparent 67% decrease in the incidence of acute HBV infection in the US between 1990 and 2000 [33]. The trend has continued, with data collected through 2006 showing that the incidence of acute hepatitis B declined by 81% to the lowest rate ever recorded (1.6 cases per 100 000 population) [34]. Declines occurred among all age groups but were greatest among children under the age of 15 (98% decline), a clear result of the recommendation and implementation of universal vaccination of children against hepatitis B. However, in adults the rate of new infections remained high, particularly among males aged 25–44 years. An estimated 46 000 new infections occurred in the US in 2006 and 43 000 in 2007 [35]. In the northern California region, this is the result of increased immigration, particularly among API, as well as of other factors including high-risk sexual behaviour, injection drug use and tattooing, combined with historically limited HBV screening and education plans.

Priority action areas targeted to API communities and other high-risk populations should include:

- Increased awareness of hepatitis B, including risk factors for transmission and measures for prevention;
- Increased awareness of the spectrum of risks related to HBV infection;
- Development of hepatitis B education and prevention strategies that are ethnicity, culture and group specific, including education that specifically targets cultural stigma and misconceptions surrounding HBV infection;
- Development of systematic programmes for HBV screening, vaccination and management;
- State-mandated training of physicians and other healthcare practitioners to provide (and permit) standards of care, education and counselling to individuals with and at risk for hepatitis B.

### Screening and diagnosis

A number of factors contribute to the low level of screening for and diagnosis of HBV infection:

- Unavailability of free or low-cost testing at community and healthcare centres;
- Lack of awareness among primary care healthcare providers of:
  - The prevalence and implications of HBV infection in the API population;
  - How to test for HBV and how to monitor those who are infected;
  - Asymptomatic status and normal liver tests among many persons who were infected at birth or during early childhood;
- Lack of understanding among at-risk individuals that HBV testing is not typically conducted routinely, and that the diagnosis can only be made when specific blood tests are conducted;
- Fear among API regarding the societal and healthcare implications of an officially recorded positive diagnosis.

The availability and accessibility of screening programmes are crucial for the diagnosis of HBV infection. There is a need to expand testing and counselling services, especially within API communities. Information regarding sites and locations for testing and vaccination should be provided in API newspapers and magazines and by local community organizations as some individuals may have language barriers. Multilingual forms and educational materials are essential. Programmes should also identify community leaders within at-risk populations who are fluent in multiple languages and can convey the requisite information in a trustworthy manner.

## Treatment and support

Several factors contribute to a low level of commitment to treat active HBV infection:

- Individuals with hepatitis B often have no symptoms and may have normal (or apparently normal) liver enzyme tests;
- Individuals have little or no appreciation for the potentially serious implications of untreated HBV infection;
- Individuals with hepatitis B and/or their physicians may be unaware that there are effective and safe treatment options;
- Individuals may be unable to afford available treatments or at least perceive them as unaffordable;
- Many individuals are not appropriately educated about the medication(s) they are taking and the importance of long-term adherence.

Several actions are required to improve and support treatment:

- Establish hepatitis B clinics within API communities;
- Ensure adequate training of physicians and other care providers, particularly those with at-risk or HBV-infected patients;
- Incentivize physicians to test for HBV in at-risk groups; as an example, the *SF Hep B Free* campaign generated and advertised a San Francisco ‘Clinician's Honor Roll’ to engender public confidence in their hepatitis B care by those clinicians who are listed; both the current Clinician's Honor Roll and the Honor Roll Pledge Form are available at: http://www.sfhepbfree.org/clinicians.
- Support primary care physicians with information on hepatitis B therapies;
- Utilize or develop educational materials for API and other at-risk groups regarding available treatments for active hepatitis B, including information on effectiveness, side effects and the importance of adherence;
- Provide free or low-cost vaccination for family members, partners and those at risk through community clinics, local hospitals and health fairs.

## Screening and surveillance

Key actions for hepatitis B screening and surveillance include:

- Development of culturally sensitive screening and surveillance strategies for:
  - API Americans;
  - Other high-risk populations;
- Administration of local screening and vaccination programmes;
- Establishment of confidential local hepatitis B databases to track and follow up with individuals who test positive for HBV on screening;
- Prevention of any form of penalty for testing positive.

## Research

An evidence base for an effective public health response to the local hepatitis B epidemic needs to be established through well-conducted research in regions such as San Francisco, the Bay Area and northern California. There is a need to dedicate public research funds to hepatitis B and to increase local research strategies in the areas of hepatitis B prevention, surveillance, treatment and pathogenesis. Primary action areas for research include:

- Local funding for strategic epidemiological research, particularly to better understand the patterns of HBV transmission, the impact of prevention strategies and the extent of liver disease burden in API American communities;
- Socioeconomic, cultural and behavioural research within API communities to identify and better understand barriers to screening and treatment;
- Basic scientific and translational research to better understand the pathogenesis of disease caused by HBV. These activities include:
- Study and correlation of HBV genetic factors with the outcome of HBV infection;
- HBV genotype information:
  - Distribution and partitioning of genotypes and subgenotypes in different populations;
  - Examination of viral mosaicism:
    - Identification of factors that lead to foster and amplify recombinant strains.
  - Comparison of substitution rates in the pre-S regions.
  - Correlation of i-iii with liver disease activation and liver cancer.
  - Treatment outcome/drug resistance rates.

- Core and precore substitution analysis in HBeAg+ cases:
  - Correlation with natural history and treatment outcomes.
- RT/POL substitution analysis:
  - Frequency in treatment-naïve cases and the effect on treatment-induced viral response kinetics and resistance rates.
- Host genetic and immunological factors and the outcome of hepatitis B:
  - A comparison of immunogenetic polymorphisms in individuals with different outcomes.
  - A study of immune responses during respective phases of disease activation.
- Factors influencing liver cancer:
  - Race and gender.
  - HBV genetic analyses.
  - Host oncogene and tumour suppressor gene analyses.
  - DNA and protein adduct measurement to examine the exposure to genotoxic and carcinogenic substances.
  - HBV-associated liver cancer biomarker assessment, validation and development.
- Establishment of a pretreatment and ‘on-treatment’ biorepository for research purposes, including assessment of the prevalence and mechanism of anti-HBV drug resistance.
- Establishment of a confidential database to track the clinical and molecular trajectories of individuals with hepatitis B.

## Call to action

The need for an integrated approach to CHB is abundantly clear. We believe that policy-makers, researchers, educators, healthcare professionals, awareness and treatment activists and community members should expeditiously accept this call to action:

- To develop and implement a targeted strategy for the increased awareness, prevention, control *and* medical management of hepatitis B in high-risk populations.
- To implement provisions for programmatic surveillance, early detection, vaccination and preventive education and training for at-risk individuals and their primary healthcare providers.
- To develop local and community-based hepatitis B testing programmes to screen high-risk populations, to provide information and education and to promote HBV testing and behaviour modification.
- To make testing and vaccination available to all adults and foreign-born adolescents, especially those from high-risk populations and ensure continuation of universal vaccination of children and adolescents.
- To provide educational programmes on hepatitis B to community-based healthcare providers, especially those who are responsible for the care of large populations of API Americans.
- To initiate community-based research programmes to evaluate best practices for hepatitis B prevention.

This landmark San Francisco HBV screening, vaccination and management programme will promote not only local and regional but also national and international awareness and can set targets for other cities in the US and potentially elsewhere. The proposed opportunistic translational research will similarly form a hub of interest for hepatologists and HBV researchers throughout the US and around the globe. The combined results of this type of integrated approach to CHB may significantly help to turn the tide against this disease, preventing infection, improving the lives of affected individuals and greatly reducing the associated healthcare burden.

## Statement of interests

Authors' declaration of personal interests: Robert Gish has received grants from and has consulting relationships with Gilead and Bristol Myers-Squibb, but no financial investment positions with any company. Stewart Cooper has no financial disclosures related to this manuscript. Declaration of funding interests: Funding for the HBV screening by the California Pacific Medical Center programme discussed in the manuscript was provided by an unrestricted charitable grant from California Pacific Medical Center Foundation.

## Abbreviations

AAHBP: Asian American Hepatitis B Program
API: Asians and Pacific Islanders
CDC: Centers for Disease Control and Prevention
CHB: chronic hepatitis B
CPMC: California Pacific Medical Center
HBF: The Hepatitis B Foundation
HBIG: hepatitis B immune globulin
HBV: hepatitis B virus
HCC: hepatocellular carcinoma
HCV: hepatitis C virus
HIV: human immunodeficiency virus
JRC: Jade Ribbon Campaign
SFDPH: San Francisco Department of Public Health
SPMF: Sutter Pacific Medical Foundation
UCSF: University of California at San Francisco
US: United States
